# Opportunities and challenges for the inclusion of patient preferences in the medical product life cycle: a systematic review

**DOI:** 10.1186/s12911-019-0875-z

**Published:** 2019-10-04

**Authors:** Rosanne Janssens, Isabelle Huys, Eline van Overbeeke, Chiara Whichello, Sarah Harding, Jürgen Kübler, Juhaeri Juhaeri, Antonio Ciaglia, Steven Simoens, Hilde Stevens, Meredith Smith, Bennett Levitan, Irina Cleemput, Esther de Bekker-Grob, Jorien Veldwijk

**Affiliations:** 1 Department of Pharmaceutical and Pharmacological Sciences, KU Leuven, Herestraat 49, Box 521, 3000 Leuven, Belgium; 2 Erasmus School of Health Policy & Management (ESHPM) and Erasmus Choice Modelling Centre (ECMC), Erasmus University Rotterdam, P.O. Box 1738, 3000 DR Rotterdam, The Netherlands; 3 Takeda International, UK Branch, 61 Aldwych, London, WC2B 4AE UK; 4 QSciCon, Europabadstr. 8, 35041 Marburg, Germany; 5 Sanofi, 55 Corporate Drive, Bridgewater Township, NJ 08807 USA; 6 International Alliance of Patients’ Organizations, 49-51 East Rd, Hoxton, London, N1 6AH UK; 7 Institute for Interdisciplinary Innovation in healthcare (I3h), Université libre de Bruxelles (ULB), Route de Lennik 808, 1070 Brussels, Belgium; 8 Amgen, Inc., Thousand Oaks, California USA; 9 Global R&D Epidemiology, Janssen Research & Development, 1125 Trenton-Harbourton Road, PO Box 200, Titusville, NJ 08560 USA; 10 Belgian Health Care Knowledge Centre (KCE), Kruidtuinlaan 55, 1000 Brussels, Belgium

**Keywords:** Patient preferences, Drug development, Drug evaluation, Decision-making, Stakeholders, Drug life cycle, Marketing authorization, Health technology assessment, Reimbursement

## Abstract

**Background:**

The inclusion of patient preferences (PP) in the medical product life cycle is a topic of growing interest to stakeholders such as academics, Health Technology Assessment (HTA) bodies, reimbursement agencies, industry, patients, physicians and regulators. This review aimed to understand the potential roles, reasons for using PP and the expectations, concerns and requirements associated with PP in industry processes, regulatory benefit-risk assessment (BRA) and marketing authorization (MA), and HTA and reimbursement decision-making.

**Methods:**

A systematic review of peer-reviewed and grey literature published between January 2011 and March 2018 was performed. Consulted databases were EconLit, Embase, Guidelines International Network, PsycINFO and PubMed. A two-step strategy was used to select literature. Literature was analyzed using NVivo (QSR international).

**Results:**

From 1015 initially identified documents, 72 were included. Most were written from an academic perspective (61%) and focused on PP in BRA/MA and/or HTA/reimbursement (73%). Using PP to improve understanding of patients’ valuations of treatment outcomes, patients’ benefit-risk trade-offs and preference heterogeneity were roles identified in all three decision-making contexts. Reasons for using PP relate to the unique insights and position of patients and the positive effect of including PP on the quality of the decision-making process. Concerns shared across decision-making contexts included methodological questions concerning the validity, reliability and cognitive burden of preference methods. In order to use PP, general, operational and quality requirements were identified, including recognition of the importance of PP and ensuring patient understanding in PP studies.

**Conclusions:**

Despite the array of opportunities and added value of using PP throughout the different steps of the MPLC identified in this review, their inclusion in decision-making is hampered by methodological challenges and lack of specific guidance on how to tackle these challenges when undertaking PP studies. To support the development of such guidance, more best practice PP studies and PP studies investigating the methodological issues identified in this review are critically needed.

**Electronic supplementary material:**

The online version of this article (10.1186/s12911-019-0875-z) contains supplementary material, which is available to authorized users.

## Background

Increasingly, the patient's perspective is considered essential on all levels of decision-making throughout the lifecycle of drugs and medical devices (i.e. the *medical product life cycle* (MPLC)) [1, 2]. This is demonstrated by a growth of literature on the roles of patients’ perspectives in drug and medical device development [3–5], regulatory *benefit-risk assessment* (BRA), *Health Technology Assessment* (HTA) [6–9] and clinical practice guideline development [10, 11]. The term ‘medical product’ will be used hereafter as an umbrella term for drugs (or human medicinal products) and medical devices as defined by the European Commission [12, 13] and the *US Food and Drug Administration* (FDA) [14].

A particular area of interest is the measurement and use of *patient preferences* (PP) [15, 16]. Although no unique definition exists for PP across research fields and disciplines [17–20], the FDA refers to PP by defining patient preference information as *“qualitative or quantitative assessments of the relative desirability or acceptability to patients of specified alternatives or choices among outcomes or other attributes*^1^ *that differ among alternative health interventions”* [14]. PP can be investigated through qualitative and/or quantitative methods [19]. While qualitative methods (e.g. interviews) generate information about patient experiences and perspectives, quantitative methods (e.g. *discrete choice experiments*) collect numerical data [21].

Despite broad interest in the measurement and application of PP, a comprehensive overview of their specific roles and reasons to use them and the expectations, concerns and requirements regarding their use in the different decision-making contexts of the MPLC is lacking. This literature review attempts to address this gap by providing an overview of their potential roles, and reasons for using PP, as well as the expectations, requirements and concerns related to their use in the following decision-making contexts of the MPLC: i) industry processes, ii) regulatory BRA and *marketing authorization* (MA), and iii) HTA and reimbursement. Insights of this review show opportunities and challenges for the use of PP in decision-making by all stakeholders involved in these decision-making contexts, thereby paving the way for patient-centric decision-making throughout the MPLC.

## Methods

### Review context

This study was conducted as part of the *Patient Preferences in Benefit-Risk Assessments during the Drug Life Cycle* (PREFER) project, a five-year project that received funding from the *Innovative Medicines Initiative* (IMI) 2 Joint Undertaking. PREFER aims to establish recommendations to guide industry, regulatory authorities and HTA/reimbursement bodies on how and when to include PP [22, 23]. While PP are gaining attention, their use in decision-making remains limited [2]. One of the first steps towards recommendations about PP was therefore to understand what hampers their current use (i.e. the challenges for their use) and what potential decisions and steps of the MPLC PP may inform (i.e. the opportunities). These questions formed the basis of the review questions.

### Review questions and search strategy

The review was guided by the following review questions, pertaining both to the preference method itself and the application of PP to decision-making: i) what roles do PP have to play in the MPLC and what are reasons to use them? (desires), ii) what is expected to happen when PP are used in the MPLC? (expectations), iii) what concerns arise for the use of PP in the MPLC? (concerns) and iv) what is needed in order to use PP in the MPLC? (requirements). Search queries were developed based upon the review questions and consisted of Medical Subject Headings terms and free text words (Additional file 1). A preliminary scoping exercise with the initially developed search queries revealed that a large part of the literature retrieved focused on the use of PP in individual treatment decision-making and/or on the use of PP in the context of monitoring and biomarkers, both of which do not form the focus of this review. Therefore, a concept related to shared decision-making, monitoring and biomarkers was combined to the search query via ‘NOT’. A research librarian from Erasmus Rotterdam University conducted the search between January 2011 and March 2018 (so that included documents reflect contemporary issues related to PP) and in the following databases: EconLit, Embase, Guidelines International Network, PsycINFO and PubMed. Peer-reviewed publications were also identified through hand searching and snowballing. All PREFER collaborators were asked to share other relevant publicly available literature (grey literature, e.g. regulatory documents or HTA reports).

**Fig. 1:**
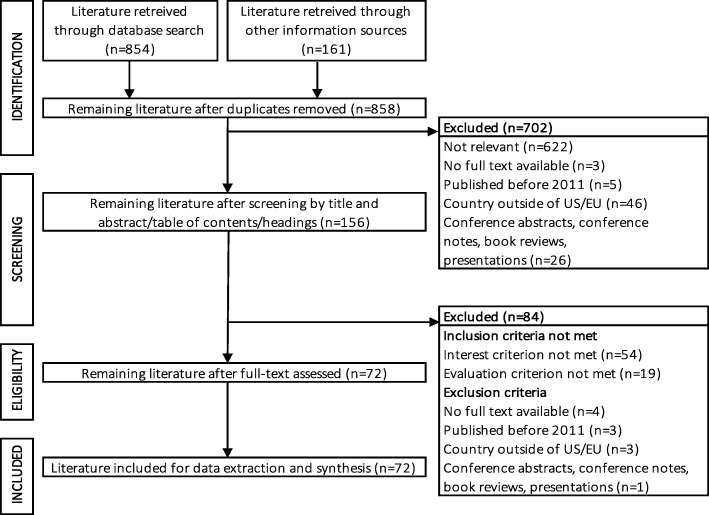
Flowchart showing the process of identifying and selecting documents for this review. Initially, 1015 documents were retrieved. From 858 non-duplicate documents, 702 were excluded, based on a screening of their title and abstract, table of contents or headings against their relevance to the review questions and exclusion criteria. An additional 84 documents were excluded after full text review against the in- and exclusion criteria, resulting in a total of 72 documents included for analysis

A two-step screening strategy was used (Fig. 1). First, title and abstract of peer-reviewed publications and the table of contents or headings of grey literature were screened for relevance to the review questions and exclusion criteria by three researchers (RJ, EvO, CW). Each document was independently screened by two researchers and disagreements were resolved by discussion. Second, full texts were screened to the in- and exclusion criteria by one researcher (RJ).

### Selection criteria

The following inclusion criteria were applied: i) literature types regulatory documents, HTA reports, project reports and workshop reports (grey literature), (systematic) reviews, original research articles (e.g. published PP studies) and perspective articles (white literature), ii) perspective: literature describing the view of at least one of the following stakeholders was included: academics, HTA/reimbursement bodies, pharmaceutical or medical device industry, patients, caregivers and patient organizations, physicians and regulatory authorities, iii) interest: included literature had to describe the use of a preference method in the MPLC. Literature describing only the use of preference methods in the context of individual treatment decision-making or clinical practice guideline development were excluded, iv) evaluation: only literature describing at least one of the proposed review questions was found eligible. The following exclusion criteria were applied: i) non-English, ii) no full text available, iii) published before 2011 (so that included documents reflect contemporary issues related to PP), iv) non-EU or non-US (in view of the scope of this study) and v) conference abstracts, conference notes, book reviews and presentations.

### Data analysis

The following steps were undertaken by one researcher (RJ) to analyze included literature: i) information on the literature type, stakeholder perspective and decision-making context was extracted (Table 1, Additional file 2), ii) a coding tree was developed to code the text (Additional file 3), iii) text was coded using the NVivo PRO 11 software (QSR international), iv) tables were developed based upon the structure of the coding tree using Microsoft Excel and these tables were subsequently used to describe literature per review question. Examples of PP studies included in the review were added to illustrate the findings.

**Table 1:** Characteristics of included literature

Characteristics of included documents (*n* = 72)	n	%
**1. Literature type**		
Original research	23	32
Review	17	24
Perspective article	11	15
Project report	7	10
Systematic review	4	6
Workshop report	3	4
Regulatory document	2	3
HTA report	2	3
Other	3	4
**2. Main decision-making context described**		
BRA/MA	25	35
HTA/reimbursement	15	21
BRA/MA + HTA/reimbursement	12	17
IPDM	5	7
ITD + BRA/MA	3	4
IPDM + HTA/reimbursement + BRA/MA	3	4
IPDM + BRA/MA	4	6
ITD + BRA/MA + HTA/reimbursement	2	3
BRA/MA + HTA/reimbursement + ITD + CPG	1	1
HTA/reimbursement + IPDM	1	1
HTA/reimbursement + CPG	1	1
**3. Stakeholder perspective**		
Academic	44	61
Regulatory authority	8	11
Industry/CRO	7	10
HTA body	3	4
Patient organization	3	4
Other	7	10

Number and percentage of included documents per literature type, main decision-making context described and stakeholder perspective (bold font). Each document was assigned to a stakeholder perspective: for primary research articles, (systematic) reviews and perspective articles, the affiliation cited of the first author was used to assign a stakeholder perspective. For regulatory documents, the regulatory authority perspective was assigned. HTA reports were assigned to the HTA body perspective. For project reports, since those are written from multiple stakeholder perspectives, they could not be assigned to a specific stakeholder perspective. *HTA* Health Technology Assessment, *BRA* benefit-risk assessment, *MA* marketing authorization, *IPDM* industry processes and decision-making, *CPG* clinical practice guideline development, *ITD* individual treatment decision-making, *CRO* contract research organization

## Results

The initial search identified 1015 documents. Seventy-two documents were included (Fig. 1, Additional file 2). Most were: i) original research (32%) or reviews (24%), ii) focused on PP in BRA/MA decision-making (35%), HTA/reimbursement (21%) or both (17%) and iii) written from an academic perspective (61%) (Table 1).

### 1. What roles do PP have to play in the MPLC and what are reasons to use them? (desires)

The potential roles of PP in the MPLC can be categorized into industry processes, regulatory BRA/MA and HTA and reimbursement (Table 2). Reasons for using PP include reasons related to the unique insights and position of patients and reasons related to the positive effect of including PP on the quality of the decision-making process (Table 3). Following the rationale that knowing how patients value treatment benefits and risks is essential because only patients know what it is like to live with their disease**,** and the idea that patients and regulators may value benefits and risks differently (Table 3), Ho et al. conducted a first PP study to inform regulatory BRA of medical devices for obese patients [45]. The authors incorporated the attribute weights resulting from the study in a tool that informs BRA for the approval of new medical devices; FDA reviewers could then compare efficacy results from clinical trials with the minimum benefit (i.e. weight loss) required as indicated by the tool [45]. Although the study was specifically designed to inform regulatory MA of new devices, they state that their results could also guide clinical trial design and post-approval decisions (Table 2).

**Table 2:** Potential roles of PP in the MPLC

**1. Potential roles of PP in industry processes**	
**1.1 Early development**	
• Informing ‘go/no-go’ decisions (e.g. internal prioritization portfolio decisions) [24]	
• Informing resource allocation decisions among multiple diseases [24]	
• Defining areas of unmet medical need [14, 16, 24]	
• Influencing which medical product will be developed [24]	
• Informing the design of a target product profile [14, 19, 27–29]	
**1.2 Clinical trial design**	
• Quantifying how clinical outcomes, benefits and risks are perceived [14, 19, 30–34]	
• Indicating which clinical endpoints are of highest importance to patients [14, 31–33, 35]	
• Indicating which endpoints should (not) be considered [31]	
• Informing enrollment criteria and sample populations [19, 31, 33]	
• Informing clinical trial sample size [27]	
• Calculating acceptable levels of uncertainty (significance level and power) [36]	
• Analyzing clinical trials [14, 19]	
• Defining subgroups with different benefit-risk trade-offs [19, 24, 37]	
**1.3 Product labelling** [14, 19, 37]	
**1.4 Post-marketing**	
• Subgroup PP information for suggesting new markets for present indications [37]	
• Subgroup PP information for pointing to specific treatment opportunities [37]	
• Informing new innovations [14]	
• Redesigning and improving existing products [14, 19]	
• Informing expanded indications or populations [14]	
• Informing risk assessments underlying product recalls [19]	
• Optimizing promotional materials [19]	
**1.5 Pharmacovigilance activities** [19, 38, 39]	
• Planning and evaluating BRAs and risk management [39]	
**2. Potential roles of PP in BRA/MA**	
• Highlighting patients’ needs for treatment [25, 26]	
• Highlighting differences in views between patients and decision-makers [19, 24, 40–42]	
• Highlighting situations with need for transparent communication about decision [42]	
• Providing quantitative measures of how patients view their choices [24]	
• Weighing (clinical) outcomes and attributes [14, 19, 25, 30, 34, 37, 38, 40, 43–48]	
• Identifying most relevant outcomes to patients [14, 19, 24, 26, 37, 48, 49]	
• Identifying outcomes with less perceived meaning [50]	
• Providing insights into patient perspectives on other aspects of treatment (e.g. dosing) [34]	
• Indicating patient benefit-risk trade-offs [18, 19, 24, 26, 34, 37, 38, 45, 47, 49, 51]	
• Indicating whether patients are likely to use therapy if approved [41]	
• Indicating how patients compare benefits and risks between treatment options [24]	
• Indicating how patients weigh benefits and risks as the disease progresses [24]	
• Enabling quantitative benefit-risk modelling in complex cases [19, 36, 37]	
• Providing information on uncertainty tolerance [24, 49]	
• Understanding patient heterogeneity [14, 19, 24, 37, 40, 42, 45, 52, 53]	
• Tailoring MA decision based on subgroups with homogeneous preferences [14, 37, 42, 45]	
**3. Potential roles of PP in HTA/reimbursement**	
• Indicating patients’ preferred treatments/technologies/healthcare services [54–57]	
• Indicating patients’ preferred health states (quality of life) [52]	
• Indicating patients’ preferred mode of administration [52, 56]	
• Indicating patients’ preferred clinical outcomes (including benefits/risks) [30, 50, 52]	
• Highlighting potential differences in views between patients and decision-makers [40]	
• Selecting, prioritizing or weighing endpoints and criteria [15, 18, 30, 44, 47, 50, 58]	
• Highlighting the value of a treatment when the QALY is considered too narrow [59]	
• Examining relative benefit-risk trade-offs [44, 54]	
• Estimating willingness to pay or willingness to accept compensation [54]	
• Predicting uptake rates [54]	
• Indicating the general acceptability of a technology to patients [19, 56, 60]	
• Providing input for economic evaluations (e.g. cost-utility analyses) [30, 47, 50, 53, 54, 61]	
• Contributing to prioritization of topics for HTA [30]	
• Identifying heterogeneity and segments of the patient population [52, 53]	
• Tailoring reimbursement decisions based upon preference heterogeneity [52]	

Potential roles of PP in the MPLC grouped per decision-making context (bold and underlined font). *PP* patient preferences, *HTA* Health Technology Assessment, *BRA* benefit-risk assessment, *MA* marketing authorization, *MPLC* medical product life cycle, *QALY* Quality Adjusted Life Years

**Table 3:** Reasons for using PP in the MPLC

**1. Reasons related to the unique insights of patients**	
• Patients have experiential knowledge of disease and treatment [16, 24, 38, 43, 54, 60, 62]	
• Decision-makers and patients might have differing preferences [19, 40, 44, 58, 63]	
• It challenges the opinions on the importance of endpoints [30, 52]	
**2. Reasons related to the unique position of patients**	
• Patients are the ultimate beneficiaries/end-consumers of healthcare [25, 31]	
• Patients are directly affected by the decision [38, 43, 53, 54, 60, 62]	
• Patients’ lives are affected by whether their concerns were considered [64]	
• Patient benefit is an objective of providing healthcare services [64]	
**3. Reasons related to the positive effect on quality of the decision-making process**	
• It enables judging the consistency of decisions with patient values [64]	
• It enables a more patient-centered decision-making [19, 36, 40, 52, 53, 58]	
• It allows evidence-based consideration of patient perspectives [24, 36, 38, 40, 43, 45, 52, 58, 64, 65]	
• It ensures patient needs are better met [25, 53, 64]	
• Measurements of clinical effects usually do not sufficiently capture PP [38, 64]	
• It facilitates integration of patient concerns into decision-making [66]	
• It increases the effectiveness of patient involvement strategies [62]	
• It solves the issue of which patients to involve directly in decision-making [38]	
• It may be more representative than direct patient involvement [24, 25, 38, 40, 43, 58, 60, 62, 67, 68]	
• It is required for the implementation of evidence-based medicine [64]	

Reasons for using PP grouped into reasons related to the unique insights and position of patients and reasons related to the positive effect of including PP on decision-making (bold and underlined font). *PP* patient preferences, *MPLC* medical product life cycle

### 2. What is expected to happen when PP are used in the MPLC? (expectations)

A range of expectations were identified in the literature review. Using PP for defining treatment attributes is expected to deliver improved health outcomes for patients [14]. PP for the selection of clinical endpoint selection is expected to: i) increase the willingness of participants to enroll in and complete a clinical trial, thereby accelerating clinical development [14, 32], ii) provide more meaningful results to future patients and iii) improve the adherence of this population with the medical product once being marketed [33, 69]. Chow et al. [33] quantified the importance of clinical trial endpoints used in cardiovascular clinical trials according to patients. They expect that, should these endpoints be selected in cardiovascular clinical trials, results from such trials would be perceived with greater validity by those reviewing the trial data [33].

Use of PP in BRA/MA and HTA/reimbursement is expected to result in: i) a higher quality decision by better alignment between the decision and patients’ values and unmet needs [25, 38, 45, 64], ii) greater legitimacy or accountability of the decision as result of taking into account clinical, social and ethical aspects of medical products that may not be considered by a professional panel of decision-makers [25], iii) an increased understanding and acceptance of the decision by the public and stakeholders because preferences of those affected by the decision were considered [25, 38, 63, 70], iv) more public trust in the decision-making process [71], and v) an increased collection of PP [64]. Finally, incorporating PP information on subgroups for whom a specific treatment will produce more benefit could increase the effectiveness and efficiency of medical products [65].

### 3. What concerns arise for the use of PP in the MPLC? (concerns)

Concerns related to using PP in the MPLC can be categorized into three types: i) general concerns (broad issues applicable to all decision-making contexts of the MPLC, e.g., lack of familiarity with preference methods among stakeholders), ii) methodological concerns (those related to measuring PP, applicable to all decision-making contexts of the MPLC, e.g., low reliability of PP studies) and iii) concerns specifically related to BRA/MA and/or HTA/reimbursement (issues related to PP specifically for a certain decision-making context, e.g., lack of clarity about how to align PP with the *Quality Adjusted Life Years* (QALY) measure in HTA/reimbursement) (Table 4).The German HTA *Institute for Quality and Efficiency in Health Care* (IQWiG) piloted the preference method *Analytical Hierarchy Process* (AHP) for the identification, weighting and prioritization of outcomes for the treatment of depression [74]. While concluding that the attribute weights resulting from their PP study could both guide industry decisions on clinical trial endpoint selection *and* HTA processes when prioritizing outcomes, they report methodological concerns such as correlating attributes and question the representativeness of the study population and transferability of the results to the entire patient population [74] (Table 4).

**Table 4:** Concerns related to PP in MPLC

**1. General concerns related to PP in industry, BRA/MA and HTA/reimbursement**	
• Lack of clarity and (regulatory) guidance about:	
○ Definition of PP, hampering communication between stakeholders [1, 62]	
○ Under what conditions to measure/use PP [1, 19]	
○ For which medical product to collect PP [19, 27, 37]	
○ When to conduct a PP study: before, during or after clinical development [19, 27, 37]	
○ What preference method to use [19, 40, 72]	
○ Which attributes to select in a PP study [19, 30, 50]	
○ How to assure validity in a PP study [19, 38]	
○ Whose preferences to measure (e.g. required disease experience) [19, 27, 44, 54, 73]	
○ How to deal with preference heterogeneity [54]	
○ Which stakeholder should collect PP [38]	
○ Who is responsible for PP results and potential biases in results [38]	
•Lack of familiarity among stakeholders with preference methods [16, 19, 24, 34]	
•Lack of patients’ knowledge and capability of expressing preferences [62]	
**2. Methodological concerns related to PP in industry, BRA/MA and HTA/reimbursement**	
• Low validity and reliability of preference methods [19, 25, 43]	
• Overlap in interpretation of attributes and interacting/overlapping attributes [30, 35, 50]	
• Tension between methodologically strong methods and their cognitive burden [18, 48]	
• Risk of neglecting of patient heterogeneity in PP studies [40, 52, 58]	
• Elicited PP are constructed and shaped by how information is presented [62]	
• Elicited PP are influenced by external factors [62]	
• Heuristics, inert or flexible preferences and measurement errors [19, 24, 27, 38, 48]	
• Challenge of communicating the quantitative health information to patients [14]	
• Innumeracy of the participants [38, 43]	
• Respondents not taking time to complete the survey of the PP study [35]	
• Lack of understanding among respondents [35]	
• Question framing in preference surveys [55]	
• Difficulty of balancing between understandability and accuracy of questions [55]	
• Ensuring representativeness of the sample [27, 50, 55]	
**3. Concerns specifically related to PP in BRA/MA and HTA/reimbursement**	
• Lack of clarity about:	
○ How PP will be used and reviewed by decision-makers [19, 24, 38]	
○ How to submit PP for BRA/MA and HTA/reimbursement [24, 53]	
○ Standards for measuring PP for informing BRA/MA and HTA/reimbursement [24, 72]	
**4. Concerns specifically related to PP in HTA/reimbursement**	
• Lack of clarity about:	
○ Measuring *patient* preferences versus *public* preferences [54, 59, 62]	
○ Measuring PP for health aspects or also for non-health aspects [1]	
○ Incorporating PP in economic evaluations or not [1]	
○ Using quantitative and/or qualitative PP in reimbursement decisions [1, 59]	
○ Where and how to incorporate PP in current procedures [1, 18, 62]	
○ How to align PP with the traditional QALY calculation [62]	
○ How to conduct a systematic review on PP studies for informing HTA [60]	
○ What weight PP should receive versus other decision criteria [1, 62]	
• Current recommendation of HTA agencies (e.g. the UK, the Netherlands) to use generic measures, whereas PP elicited via PP studies are often condition-specific [59]	
• Current use of cost-utility analysis, which does not require quantitative PP beyond health state utilities [59]	
• Low generalizability of PP study results when characteristics of healthcare system are being valued as these characteristics are often system-, country- or culture-specific [55, 62]	
• Time, funding and staff required for incorporating PP in HTA/reimbursement [1]	

Concerns related to using PP in the MPLC grouped according to their nature and the decision-making context they apply to: general concerns, methodological concerns and concerns specifically related to BRA/MA and/or HTA/reimbursement (bold and underlined font). *PP* patient preferences, *HTA* Health Technology Assessment, *BRA* benefit-risk assessment, *MA* marketing authorization, *MPLC* medical product life cycle, *QALY* Quality Adjusted Life Years

### 4. What is needed in order to use PP in the MPLC? (requirements)

Requirements related to using PP across the different decision-making contexts of the MPLC can be categorized into: i) general requirements (broad aspects that are needed to measure and use PP, e.g. guidance on PP studies), ii) operational requirements (non-methodological prerequisites related to the execution of PP studies, e.g. regarding the timing of a PP study) and iii) quality requirements (prerequisites that increase the quality of the PP study, e.g. study objectivity) (Table 5). Based on their experience from quantifying benefit-risk preferences among rare disease patients and caregivers, Morel et al. [51] conclude that while researchers of novel medical products for rare diseases should be encouraged to invest in use of preference methods, specific regulatory guidance is needed to acknowledge the importance of PP and to state when in the MPLC preference methods should be used (Table 5).

**Table 5:** Requirements related to PP in the MPLC

**1. General requirements**	
• Recognition of the value of PP among stakeholders [24, 25, 39, 51, 59, 64]	
• Consensus on the role of PP in decision-making [1, 62]	
• More familiarity among stakeholders with PP studies [19, 34, 48, 66, 70, 72]	
• More educated researchers in preference research [53]	
• Resources to evaluate PP [1, 48]	
• Taxonomic work for PP research [1, 60]	
• Guidance on:	
○ When during development to measure PP [1, 34, 51]	
○ Which preference method to use in which circumstance [1, 40, 44, 56, 72]	
○ Whose preferences to measure (e.g. required disease experience) [1, 19, 44]	
○ Sample size [37]	
○ Good research practice and quality criteria for PP studies [1, 19, 38, 43, 44, 62]	
○ How to ensure validity of a PP study [75]	
○ How to report about PP studies [44]	
• Further research to:	
○ Validate and test preference methods [37, 44, 46, 66, 76]	
○ Identify methods for integrating clinical evidence in PP study analysis [50, 56]	
○ Investigate methodological issues (e.g. hindsight bias) [62]	
○ Compare the performance of different methods in a given situation [37]	
○ Determine impact of changing list of attributes with any given method [37]	
○ Explore statistical methods to detect preference heterogeneity [77]	
○ Guide the development of newer methods for eliciting PP [76]	
○ Assess comprehension differences by participants between methods [76]	
○ Assess impact of the level of previous education on PP [33]	
○ Quantify the effect of the attribute descriptions on elicited PP [78]	
**2. Operational requirements**	
• Requirements related to timing of PP study:	
○ Decision depends on level of information of the treatments’ key risks [19]	
○ Timing needs to be decided by sponsor [19]	
○ During marketing phase to assess long-term side effects and burden [1]	
• Requirements related to dealing with PP study results:	
○ Stakeholders should be prepared for disappointing PP study results [24]	
○ PP study results should be provided to patient community and public [24]	
○ Presentation of PP study results should be tailored to the audience [79]	
○ PP study results should be described transparently [56, 75]	
**3. Quality requirements**	
• General requirements regarding design, set-up and conduct of PP studies:	
○ Selected research question should be answerable with PP study [75]	
○ Study objectivity throughout PP study [24]	
○ Independent design as design can influence analysis outcomes [25]	
○ Extensive and forward planning [19, 27, 48]	
○ Determination of objectives and attributes before design [24]	
○ Design based on prior literature and preference information [19]	
○ Clear definition of the patient sample and characteristics [19, 24, 49]	
○ Training partners on methodology, objectives and expectations of study [79]	
○ Good communication and documentation of changes to study plans [79]	
○ Methodological expertise when designing and executing a PP study [24, 70]	
○ Multi-stakeholder partnerships (patients, academics, industry) [24, 37, 79]	
○ Interaction between decision-makers and industry in design [14, 19, 24]	
○ Involvement of patients, caregivers and patient organizations [24, 42, 49, 51]	
○ Application of ‘good science’ principles [1, 19, 24, 51]	
○ Consideration of patient heterogeneity and cognitive burden [14, 40, 58, 75]	
○ Consideration of internal and external validity [75]	
○ Administration of survey by trained researchers [14]	
○ Provision of tutorial for participants if self-administered survey is used [14]	
○ Training of participants in elicitation tasks [40]	
○ Ensuring participants’ understanding of aim and how results will be used [40]	
○ Consideration of low level of health numeracy in general population [43]	
• Sample requirements:	
○ Sample should be heterogeneous (large samples, setting quotas) [19, 49, 75]	
○ Sample should be representative of population of interest [14, 19]	
○ If not possible to elicit from patients, include proxies [19, 34]	
○ Sample ideally is clinical trial population [71]	
○ Sample ideally is broader population than clinical trial population [41]	
○ Patient should be the focus, not health care professional [14]	
○ Sample should be representative of affected patients [56]	
○ Sample should be representative of target population [75]	
○ Sample that can yield reliable results should be drawn [24]	
○ PP should come from the same population as data of effectiveness [1]	
○ Both patients in remission as well as patients in recovery should be included [50]	
○ Sampling should consider sociodemographic and disease characteristics [50, 61]	
• Sample size requirements:	
○ *Adequate* size so that results are generalizable to population of interest [14]	
○ *Sufficient* size to generate acceptably robust results [24]	
○ If subgroups: *sufficient* number in each subgroup [14]	
• PP results requirements:	
○ Type of PP (qualitative vs quantitative) depends on stage and decision-making context of MPLC [1, 14, 16, 19, 60]	
○ Type of PP should be determined by research question [19]	
○ Clinical data should be collected and used to augment PP data [42, 43]	
○ Patient’s willingness *and* unwillingness to accept risks should be measured [14]	
• Preference method requirements:	
○ Method should be selected based on factors [1, 19, 40, 44, 76, 80]	
○ Method should adhere to utility theory [18, 76]	
○ Method should account for patient-relevant attributes/outcome measures [18]	
○ Methods should be easy and simple for patients to understand [18]	
• Requirements regarding attribute selection:	
○ Research question should guide attribute and level selection [75]	
○ Attributes should be broader than clinical attributes to elicit meaningful trade-offs [41]	
○ Attributes should be patient-centered to investigate meaningful attributes [49]	
○ Attributes should come from existing clinical trials [50, 81]	
○ Selection by literature, qualitative study, asking group of medical experts or decision-makers [19]	
○ Patient representatives, patients and experts should inform selection [50, 62]	
○ Attributes should not overlap [30, 35, 50]	
• Requirements regarding survey instrument:	
○ Survey should be developed with input from multiple stakeholders [24]	
○ Survey should be piloted [24, 40]	
○ Survey should include screening questions, informed consent provisions, background information, training and definitions, testing, survey questions, follow-up survey questions [24]	
○ Benefit descriptions and effectiveness measures should be carefully defined [78]	
○ Patients should understand objective of the elicitation tasks and how data will be used [40]	
○ Questions have to be asked in an open and understandable way [18, 56, 75]	
○ For choice-based preference measures, options should:	
■ Be clearly described [56]	
■ Have realistic advantages and disadvantages [56]	
■ Be communicated to patients together with their characteristics [80]	
• Requirements regarding the analysis:	
○ Interpretation of results should consider the mode of sampling [68]	
○ Interpretation of study results should be validated with patients [40]	
○ Results should be considered with preferences from other stakeholders (clinicians, decision-makers) [68]	
○ Appropriate stakeholders should interpret analysis [79]	
○ Sources of uncertainty should be reported through confidence interval and/or standard error [14]	
○ Written agreements about intellectual property and data use are needed [24]	

Requirements related to using PP in the MPLC grouped according to their type and nature: general requirements, operational requirements and quality requirements (bold and underlined font). *PP* patient preferences, *MPLC* medical product life cycle

## Discussion

Using a systematic approach, this review identified the potential roles and reasons to use PP (desires), as well as the expectations, concerns and requirements regarding their use across industry processes, BRA/MA, and HTA/reimbursement decision-making.

The three potential roles that were identified in all three decision-making contexts involved the use of PP to increase understanding of: i) how patients value (clinical) outcomes of a medical product, ii) how patients make the trade-off between benefits and risks and iii) how preferences may differ across patient subgroups (preference heterogeneity). This finding raises the question of whether a single PP study with the primary objectives of investigating these three issues could inform all three decision-making contexts and address the needs respectively, of industry, HTA/reimbursement and regulatory BRA/MA stakeholders. One could for example imagine a PP study consisting of: i) a qualitative phase, where patients are asked openly about what their needs are regarding treatment for their disease and what treatment attributes they find important, and ii) a quantitative phase, where patients are asked to choose between hypothetical treatment options that differ in how they perform on these treatment attributes. Both the results from the qualitative phase as well as the selected attributes and attribute weights derived from the quantitative phase could assist industry in: i) developing a medical product that targeted patient needs and the attributes that patients found most important and ii) subsequently selecting those clinical trial endpoints based upon the attributes patients indicated as most relevant. The selected attributes and the attribute values from the quantitative phase could also be used by regulators and HTA/reimbursement decision-makers to assess the clinical relevance of the outcomes of a medical product being evaluated for MA and reimbursement for the disease of the included patients in the PP study. Furthermore, the attribute values could be used to calculate the minimum required benefit (the minimum benefit respondents expect in order to tolerate a specific level of risk) and the maximum acceptable risk (the maximum risk respondents are willing to tolerate for a given benefit). This minimum required benefit could be used as a reference by regulatory and HTA/reimbursement decision-makers, to evaluate whether or not the clinical benefit of the medical product, as demonstrated in clinical trials exceeds this value, i.e. whether patients would accept the risks of the product under evaluation in return for its benefits. If these calculations indicated that a subgroup of patients accepted the risks in exchange for the benefit, this could inform regulators and HTA/reimbursement decision-makers on the marketing authorization or reimbursement for that subset of patients respectively. These preferred outcomes could also be incorporated in a novel *patient relevant outcome* (PRO) instrument as, for example, explained by Evers et al. [32], that could be used *during* clinical trials to evaluate how the medical product in clinical development performed on these PROs. Results from such a hypothetical clinical trial could then inform: i) regulators and HTA decision-makers regarding the performance of that medical product in terms of those PROs as observed during clinical development and ii) the developer of that medical product on how to redesign and improve the medical product in subsequent development. After the MA and reimbursement decision, the PRO instrument could be used to assess how the medical product performs on these PROs outside the clinical trial environment. This information could then inform industry on product redesign and regulatory and HTA/reimbursement stakeholders on continuation of MA and reimbursement. Finally, if these PRO measurements indicate that the medical product only performs well for a subset of patients outside the clinical trial environment this could inform continuation of MA and reimbursement for that subset of patients only.

Despite the array of potential opportunities for the use of PP in the MPLC listed in Table 2, there are few published examples of the actual use of PP study results in industry, regulatory and HTA decisions [45, 82]. As highlighted in Tables 4 and 5, a number of concerns and gaps need to be addressed in order to advance the measurement and use of PP in these decisions. More specifically, efforts need to focus on providing and encouraging: i) recognition of the importance of including PP in industry, regulatory and HTA decisions, ii) guidance on when and how to measure PP aiming to inform these stakeholder decisions and iii) increased familiarity with performing and evaluating PP studies. To promote the development of guidance, more best practice PP studies and more PP studies investigating the methodological concerns this review identified are needed on such questions as: i) how to select, apply and validate different preference methods, ii) how to choose a representative sample in order to satisfy the needs of different stakeholders and iii) how to increase understanding of the reliability and cognitive burden of different preference methods.

Although efforts to address methodological issues are crucial, they are not sufficient alone. Efforts are also needed to address how results from a PP study could be incorporated and aligned with current decision-making processes. More clarity on how results from PP studies would be used in regulatory and HTA decisions together with guidance on how such studies should be conducted could motivate stakeholders to conduct and submit a PP study (Table 5). Table 5 highlights additional concerns regarding the use of PP in HTA/reimbursement. Among the multiple ways in which PP could inform HTA/reimbursement (Table 2), the potential role of PP to inform QALY calculations in countries with publicly funded healthcare is under ongoing debate. As in these countries, HTA guides the allocation of public resources (including but not limited to patients only), it is unclear whether *public* versus *patient* preferences should be used. Incorporation of PP in HTA/reimbursement bodies of such countries would therefore not only require solving methodological questions, but also structural and political discussions on the current HTA process in those countries.

This review identified numerous operational and quality requirements involved in performing and evaluating PP studies (Table 5), including: i) ensuring a transparent description of PP study and results when communicating about the study, ii) applying the principles of ‘good science’ when conducting a PP study, iii) paying attention to the heterogeneity of PP and cognitive challenges of preference methods, iv) ensuring patient understanding of the questions asked in the PP study and v) ensuring a representative and diverse sample.

Remarkably, only 18% of the included documents focused on the use of PP in industry processes, leading to sparse results on the use PP in industry processes. As a result, further research is warranted regarding the use of PP for industry purposes, e.g. by consulting sources other than the peer-reviewed literature, including those sources that use qualitative methods such as interviews or focus groups. It was beyond the scope and aim of this review to: i) explain reasons why certain concerns or requirements exist and ii) grade the results (e.g. the identified concerns and requirements) into a hierarchy indicating their importance. Therefore, research aiming to address these issues could complement the current review, e.g. by using qualitative methods to explain the reasoning behind certain issues or differences or by using quantitative methods aimed at ranking and prioritizing the identified requirements and concerns. No literature was found that described the patient’s, caregiver’s, reimbursement agency’s or (practicing) physician’s perspective, which may have led toward a more methodological and scientific result. Therefore, research aiming to assess the perspectives of these different stakeholder groups on the measurement and the use of PP in the MPLC would complement the current review.

The main strengths of this review are its comprehensiveness and novelty in using a systematic approach to search and identify literature relevant to this topic; to the best of the authors’ knowledge, this is the first systematic review that provides an overview of the specific roles and the expectations, concerns and requirements associated with using PP in different decision-making contexts across the MPLC and for different stakeholders, including industry, BRA/MA, and HTA/reimbursement. This review also has limitations. The selection criteria led to the exclusion of: i) literature focused only on PP within individual treatment decision-making, ii) non-English literature, iii) literature from outside the US/EU and iv) literature that did not explicitly mention the use of preference methods. These criteria might have resulted in the exclusion of literature dealing with issues relevant to this review. Further, the broad time span of included literature may be viewed as a limitation since the described concerns and requirements mentioned in earlier published work might at this time already be (partially) resolved and therefore this information might not be as accurate as more recently published studies. During coding, it was sometimes unclear to what specific decision-making context a particular piece of text pertained or whether a particular piece of text needed to be coded as a: i) desire or expectation or ii) concern or a need. This difficulty resulted in this text being coded into multiple domains, which in turn led to some of the results being repeated.

## Conclusions

This review highlights the numerous opportunities for using PP in industry, BRA/MA and HTA/reimbursement decisions, from early development decisions through pharmacovigilance activities and post-marketing decisions. However, exploiting the full potential of PP in these decision-making contexts is currently hampered by remaining methodological challenges and lack of specific (regulatory) guidance on how to address these challenges when designing and performing PP studies aiming to inform decisions. To support the development of such guidance, more best practice PP studies and PP studies investigating these methodological issues are critically needed.

## Additional files

Additional file 1: Search queries (DOCX 98 kb)

Additional file 2: Included literature (DOCX 193 kb)

Additional file 3: Coding tree (DOCX 96 kb)

## Data Availability

The data that support the findings of this study are available from the corresponding author on reasonable request.

## Abbreviations

AHP: Analytical Hierarchy Process
BRA: Benefit-risk assessment
CPG: Clinical practice guideline development
CRO: Contract research organization
FDA: US Food and Drug Administration
HTA: Health Technology Assessment
IMI: Innovative Medicines Initiative
IPDM: Industry processes and decision-making
IQWiG: Institute for Quality and Efficiency in Health Care
ITD: Individual treatment decision-making
MA: Marketing authorization
MPLC: Medical product life cycle
PP: Patient preferences
PREFER: Patient Preferences in Benefit-Risk Assessments during the Drug Life Cycle
PRO: Patient relevant outcome
QALY: Quality Adjusted Life Years

## Footnotes

1 Synonyms for attributes in literature are “characteristics”, “features”, “objects” or “criteria”.
