# Supplementary material for: Opportunities and challenges for the inclusion of patient preferences in the medical product life cycle: a systematic review
Source: BMC Med Inform Decis Mak. 2019 Oct 4;19:189. doi: 10.1186/s12911-019-0875-z (PMC6778383; doi:10.1186/s12911-019-0875-z)
Supplement: Supplementary file 1 — Search queries (DOCX 98 kb) [file 12911_2019_875_MOESM1_ESM.docx]

### Additional file 1: search queries

*PubMed*

(((opinion[tiab] OR opinions[tiab] OR belief[tiab] OR beliefs[tiab] OR believes[tiab] OR view[tiab] OR views[tiab] OR viewpoint[tiab] OR viewpoints[tiab] OR attitude[tiab] OR attitudes[tiab] OR assumption[tiab] OR assumptions[tiab] OR judgment[tiab] OR judgments[tiab] OR "point of view"[tiab] OR "points of view"[tiab] OR perspective[tiab] ndiOR perspectives[tiab] OR desire[tiab] OR desires[tiab] OR expectations[tiab] OR expectation[tiab] OR prediction[tiab] OR predictions[tiab] OR outlook[tiab] OR prospect[tiab] OR prospects[tiab] OR expectancy[tiab] OR expectancies[tiab] OR concern[tiab] OR concerns[tiab] OR doubt[tiab] OR doubts[tiab] OR requirement[tiab] OR requirements[tiab] OR necessity[tiab] OR precondition[tiab] OR prerequisite[tiab] OR condition[tiab] OR demand[tiab] OR demands[tiab] OR needs[tiab] OR need[tiab] OR consideration[tiab] OR considerations[tiab] OR problem[tiab] OR problems[tiab] OR issue[tiab] OR issues[tiab] OR recommendation[tiab] OR guideline[tiab] OR recommendations[tiab] OR guidelines[tiab] OR assessment[tiab] OR assessments[tiab] OR criteria[tiab] OR criterias[tiab] OR criterion[tiab] OR "decision making"[tiab] OR "decision-making"[tiab] OR "decision point"[tiab]) AND (patient[tiab] OR patients[tiab] OR "patient advisory"[tiab] OR "patient advocacy"[tiab] OR "patient advocate"[tiab] OR "patient advocacy"[MesH] OR "patient association"[tiab] OR "patient associations"[tiab] OR "patient organization"[tiab] OR "patient organisation"[tiab] OR "patient organizations"[tiab] OR "patient organisations"[tiab] OR "health technology assessment body"[tiab] OR "health technology assessment institution"[tiab] OR "HTA body"[tiab] OR "HTA institution"[tiab] OR "reimbursement body"[tiab] OR "national reimbursement body"[tiab] OR "national reimbursement institution"[tiab] OR "reimbursement institution"[tiab] OR "reimbursement agency"[tiab] OR regulatory[tiab] OR "ministry of health"[tiab] OR "regulatory body"[tiab] OR "regulatory bodies"[tiab] OR "regulator"[tiab] OR EMA[tiab] OR "European Medicines Agency"[tiab] OR FDA[tiab] OR "Food and Drug Administration"[tiab] OR "policy maker"[tiab] OR "Health Planning Organization"[tiab] OR "Health Planning Organizations"[tiab] OR "Health Planning Organisation"[tiab] OR "Health Planning Organisations"[tiab] OR "drug industry"[tiab] OR "Medical device industry"[tiab] OR "pharmaceutical industry"[tiab] OR "pharmaceutical company"[tiab] OR "pharmaceutical companies"[tiab] OR "pharmaceutical sector"[tiab] OR "drug industry"[MesH] OR physician[tiab] OR physicians[tiab] OR clinician[tiab] OR clinicians[tiab] OR "health care professional"[tiab] OR "health care professionals"[tiab] OR "healthcare professional"[tiab] OR "healthcare professionals"[tiab] OR "health care provider"[tiab] OR "health care providers"[tiab] OR "healthcare provider"[tiab] OR "healthcare providers"[tiab] OR doctor[tiab] OR doctors[tiab] OR caregiver[tiab] OR caregivers[tiab] OR caretaker[tiab] OR caretakers[tiab] OR academic[tiab] OR academics[tiab] OR academician[tiab] OR academicians[tiab] OR researcher[tiab] OR researchers[tiab]) AND ("elicitation methods"[tiab] OR method[tiab] OR methodology[tiab] OR empirical[tiab] OR "qualitative method"[tiab] OR qualitative[tiab] OR "quantitative method"[tiab] OR quantitative[tiab] OR technique[tiab] OR techniques[tiab] OR Methods[MesH] OR Investigative Techniques/methods[MesH] OR Measuring[tiab] OR measurement[tiab] OR measurements[tiab] OR assessment[tiab] OR assessments[tiab] OR inclusion[tiab] OR including[tiab] OR include[tiab] OR incorporate[tiab] OR incorporating[tiab] OR incorporation[tiab] OR involving[tiab] OR involvement[tiab] OR involve[tiab]) AND ("life cycle of a drug"[tiab] OR "life cycle of a medical device"[tiab] OR "medical device life cycle"[tiab] OR "lifecycle of a drug"[tiab] OR "lifecycle of a medical device"[tiab] OR "medical device lifecycle"[tiab] OR "drug life cycle"[tiab] OR "drug lifecycle"[tiab] OR "drug development"[tiab] OR "medical device development"[tiab] OR "development of drugs"[tiab] OR "development of a drug"[tiab] OR "development of medical devices"[tiab] OR "development of a medical device"[tiab] OR "benefit and risk"[tiab] OR "risk and benefit"[tiab] OR "risk assessment"[MeSH] OR benefit-risk[tiab] OR risk-benefit[tiab] OR reimbursement[tiab] OR "drug research"[tiab] OR "medical device research"[tiab] OR "clinical trials"[tiab] OR "clinical trial"[tiab] OR "health technology assessment"[tiab] OR "health technology assessments"[tiab]) AND ("patients preference"[tiab] OR "patients preferences"[tiab] OR "preference of patients"[tiab] OR "preferences of patients"[tiab] OR "patient preference"[tiab] OR "patient preferences"[tiab] OR "preference of a patient"[tiab] OR "preference of the patient"[tiab] OR "preferences of a patient"[tiab] OR "preferences of the patient"[tiab] OR "patient preference"[Mesh])) NOT ("shared decision making"[tiab] OR "shared decision-making"[tiab] OR monitoring[tiab] OR biomarker[tiab] OR biomarkers[tiab]) AND ("2011/01/01"[Date - Publication] : "2018/03/31"[Date - Publication])) AND English[Language]) AND Full text[Filter]))

*Embase*

(opinion:ti,ab OR opinions:ti,ab OR belief:ti,ab OR beliefs:ti,ab OR believes:ti,ab OR view:ti,ab OR views:ti,ab OR viewpoint:ti,ab OR viewpoints:ti,ab OR attitude:ti,ab OR attitudes:ti,ab OR assumption:ti,ab OR assumptions:ti,ab OR judgment:ti,ab OR judgments:ti,ab OR 'point of view':ti,ab OR 'points of view':ti,ab OR perspective:ti,ab OR perspectives:ti,ab OR desire:ti,ab OR desires:ti,ab OR expectations:ti,ab OR expectation:ti,ab OR prediction:ti,ab OR predictions:ti,ab OR outlook:ti,ab OR prospect:ti,ab OR prospects:ti,ab OR expectancy:ti,ab OR expectancies:ti,ab OR concern:ti,ab OR concerns:ti,ab OR doubt:ti,ab OR doubts:ti,ab OR requirement:ti,ab OR requirements:ti,ab OR necessity:ti,ab OR precondition:ti,ab OR prerequisite:ti,ab OR condition:ti,ab OR demand:ti,ab OR demands:ti,ab OR needs:ti,ab OR need:ti,ab OR consideration:ti,ab OR considerations:ti,ab OR problem:ti,ab OR problems:ti,ab OR issue:ti,ab OR issues:ti,ab OR recommendation:ti,ab OR guideline:ti,ab OR recommendations:ti,ab OR guidelines:ti,ab OR assessment:ti,ab OR assessments:ti,ab OR criteria:ti,ab OR criterias:ti,ab OR criterion:ti,ab OR 'decision making':ti,ab OR 'decision-making':ti,ab OR 'decision point':ti,ab) AND (patient:ti,ab OR patients:ti,ab OR 'patient advisory':ti,ab OR 'patient advocacy':ti,ab OR 'patient advocate':ti,ab OR 'patient advocacy'/exp OR 'patient advocacy'/de OR 'patient association':ti,ab OR 'patient associations':ti,ab OR 'patient organization':ti,ab OR 'patient organisation':ti,ab OR 'patient organizations':ti,ab OR 'patient organisations':ti,ab OR 'health technology assessment body':ti,ab OR 'health technology assessment institution':ti,ab OR 'hta body':ti,ab OR 'hta institution':ti,ab OR 'reimbursement body':ti,ab OR 'national reimbursement body':ti,ab OR 'national reimbursement institution':ti,ab OR 'reimbursement institution':ti,ab OR 'reimbursement agency':ti,ab OR regulatory:ti,ab OR 'ministry of health':ti,ab OR 'regulatory body':ti,ab OR 'regulatory bodies':ti,ab OR 'regulator':ti,ab OR ema:ti,ab OR 'european medicines agency':ti,ab OR fda:ti,ab OR 'food and drug administration':ti,ab OR 'policy maker':ti,ab OR 'health planning organization':ti,ab OR 'health planning organizations':ti,ab OR 'health planning organisation':ti,ab OR 'health planning organisations':ti,ab OR 'drug industry':ti,ab OR 'medical device industry':ti,ab OR 'pharmaceutical industry':ti,ab OR 'pharmaceutical company':ti,ab OR 'pharmaceutical companies':ti,ab OR 'pharmaceutical sector':ti,ab OR 'drug industry'/exp OR 'drug industry'/de OR physician:ti,ab OR physicians:ti,ab OR clinician:ti,ab OR clinicians:ti,ab OR 'health care professional':ti,ab OR 'health care professionals':ti,ab OR 'healthcare professional':ti,ab OR 'healthcare professionals':ti,ab OR 'health care provider':ti,ab OR 'health care providers':ti,ab OR 'healthcare provider':ti,ab OR 'healthcare providers':ti,ab OR doctor:ti,ab OR doctors:ti,ab OR caregiver:ti,ab OR caregivers:ti,ab OR caretaker:ti,ab OR caretakers:ti,ab OR academic:ti,ab OR academics:ti,ab OR academician:ti,ab OR academicians:ti,ab OR researcher:ti,ab OR researchers:ti,ab) AND ('patients preference':ti,ab OR 'patients preferences':ti,ab OR 'preference of patients':ti,ab OR 'preferences of patients':ti,ab OR 'patient preference':ti,ab OR 'patient preferences':ti,ab OR 'preference of a patient':ti,ab OR 'preference of the patient':ti,ab OR 'preferences of a patient':ti,ab OR 'preferences of the patient':ti,ab OR 'patient preference'/exp OR 'patient preference'/de) AND ('elicitation methods':ti,ab OR method:ti,ab OR methodology:ti,ab OR empirical:ti,ab OR 'qualitative method':ti,ab OR qualitative:ti,ab OR 'quantitative method':ti,ab OR quantitative:ti,ab OR technique:ti,ab OR techniques:ti,ab OR 'methodology'/exp OR 'methodology'/de OR measuring:ti,ab OR measurement:ti,ab OR measurements:ti,ab OR assessment:ti,ab OR assessments:ti,ab OR inclusion:ti,ab OR including:ti,ab OR include:ti,ab OR incorporate:ti,ab OR incorporating:ti,ab OR incorporation:ti,ab OR involving:ti,ab OR involvement:ti,ab OR involve:ti,ab) AND ('life cycle of a drug':ti,ab OR 'life cycle of a medical device':ti,ab OR 'medical device life cycle':ti,ab OR 'lifecycle of a drug':ti,ab OR 'lifecycle of a medical device':ti,ab OR 'medical device lifecycle':ti,ab OR 'drug life cycle':ti,ab OR 'drug lifecycle':ti,ab OR 'drug development':ti,ab OR 'medical device development':ti,ab OR 'development of drugs':ti,ab OR 'development of a drug':ti,ab OR 'development of medical devices':ti,ab OR 'development of a medical device':ti,ab OR 'benefit and risk':ti,ab OR 'risk and benefit':ti,ab OR 'risk assessment'/exp OR 'risk assessment'/de OR 'benefit risk':ti,ab OR 'risk benefit':ti,ab OR reimbursement:ti,ab OR 'drug research':ti,ab OR 'medical device research':ti,ab OR 'clinical trials':ti,ab OR 'clinical trial':ti,ab OR 'health technology assessment':ti,ab OR 'health technology assessments':ti,ab) NOT ('shared decision making':ti,ab OR 'shared decision-making':ti,ab OR monitoring:ti,ab OR biomarker:ti,ab OR biomarkers:ti,ab) AND [english]/lim AND [embase]/lim NOT ([conference abstract]/lim OR [conference paper]/lim OR [conference review]/lim OR [editorial]/lim OR [erratum]/lim OR [letter]/lim OR [note]/lim) AND [1-1-2011]/sd NOT [1-4-2018]/sd

***PsycINFO***

((opinion or opinions or belief or beliefs or believes or view or views or viewpoint or viewpoints or attitude or attitudes or assumption or assumptions or judgment or judgments or "point of view" or "points of view" or perspective or perspectives or desire or desires or expectations or expectation or prediction or predictions or outlook or prospect or prospects or expectancy or expectancies or concern or concerns or doubt or doubts or requirement or requirements or necessity or precondition or prerequisite or condition or demand or demands or needs or need or consideration or considerations or problem or problems or issue or issues or recommendation or guideline or recommendations or guidelines or assessment or assessments or criteria or criterias or criterion or "decision making" or "decision-making" or "decision point").ab,ti. and ((patient or patients or "patient advisory" or "patient advocacy" or "patient advocate" or "patient association" or "patient associations" or "patient organization" or "patient organisation" or "patient organizations" or "patient organisations" or "health technology assessment body" or "health technology assessment institution" or "HTA body" or "HTA institution" or "reimbursement body" or "national reimbursement body" or "national reimbursement institution" or "reimbursement institution" or "reimbursement agency" or regulatory or "ministry of health" or "regulatory body" or "regulatory bodies" or "regulator" or EMA or "European Medicines Agency" or FDA or "Food and Drug Administration" or "policy maker" or "Health Planning Organization" or "Health Planning Organizations" or "Health Planning Organisation" or "Health Planning Organisations" or "drug industry" or "Medical device industry" or "pharmaceutical industry" or "pharmaceutical company" or "pharmaceutical companies" or "pharmaceutical sector").ab,ti. or pharmaceutical industry/ or physician.ab,ti. or physicians.ab,ti. or clinician.ab,ti. or clinicians.ab,ti. or "health care professional".ab,ti. or "health care professionals".ab,ti. or "healthcare professional".ab,ti. or "healthcare professionals".ab,ti. or "health care provider".ab,ti. or "health care providers".ab,ti. or "healthcare provider".ab,ti. or "healthcare providers".ab,ti. or doctor.ab,ti. or doctors.ab,ti. or caregiver.ab,ti. or caregivers.ab,ti. or caretaker.ab,ti. or caretakers.ab,ti. or academic.ab,ti. or academics.ab,ti. or academician.ab,ti. or academicians.ab,ti. or researcher.ab,ti. or researchers.ab,ti.) and ("patients preference" or "patients preferences" or "preference of patients" or "preferences of patients" or "patient preference" or "patient preferences" or "preference of a patient" or "preference of the patient" or "preferences of a patient" or "preferences of the patient").ab,ti. and (("elicitation methods" or method or methodology or empirical or "qualitative method" or qualitative or "quantitative method" or quantitative or technique or techniques or Methodology).ab,ti. or methodology/ or Measuring.ab,ti. or measurement.ab,ti. or measurements.ab,ti. or assessment.ab,ti. or assessments.ab,ti. or inclusion.ab,ti. or including.ab,ti. or include.ab,ti. or incorporate.ab,ti. or incorporating.ab,ti. or incorporation.ab,ti. or involving.ab,ti. or involvement.ab,ti. or involve.ab,ti.) and ("life cycle of a drug" or "life cycle of a medical device" or "medical device life cycle" or "lifecycle of a drug" or "lifecycle of a medical device" or "medical device lifecycle" or "drug life cycle" or "drug lifecycle" or "drug development" or "medical device development" or "development of drugs" or "development of a drug" or "development of medical devices" or "development of a medical device" or "benefit and risk" or "risk and benefit" or benefit-risk or risk-benefit or reimbursement or "drug research" or "medical device research" or "clinical trials" or "clinical trial" or "health technology assessment" or "health technology assessments").ab,ti.) not ("shared decision making" or "shared decision-making" or monitoring or biomarker or biomarkers).ab,ti. AND english.lg. AND ("2011" or "2012" or "2013" or "2014" or "2015" or "2016" or "2017" or "2018").yr.

***EconLit***

(((ti(opinion OR opinions OR belief OR beliefs OR believes OR view OR views OR viewpoint OR viewpoints OR attitude OR attitudes OR assumption OR assumptions OR judgment OR judgments OR "point of view" OR "points of view" OR perspective OR perspectives OR desire OR desires OR expectations OR expectation OR prediction OR predictions OR outlook OR prospect OR prospects OR expectancy OR expectancies OR concern OR concerns OR doubt OR doubts OR requirement OR requirements OR necessity OR precondition OR prerequisite OR condition OR demand OR demands OR needs OR need OR consideration OR considerations OR problem OR problems OR issue OR issues OR recommendation OR guideline OR recommendations OR guidelines OR assessment OR assessments OR criteria OR criterias OR criterion OR "decision making" OR "decision-making" OR "decision point") OR ab(opinion OR opinions OR belief OR beliefs OR believes OR view OR views OR viewpoint OR viewpoints OR attitude OR attitudes OR assumption OR assumptions OR judgment OR judgments OR "point of view" OR "points of view" OR perspective OR perspectives OR desire OR desires OR expectations OR expectation OR prediction OR predictions OR outlook OR prospect OR prospects OR expectancy OR expectancies OR concern OR concerns OR doubt OR doubts OR requirement OR requirements OR necessity OR precondition OR prerequisite OR condition OR demand OR demands OR needs OR need OR consideration OR considerations OR problem OR problems OR issue OR issues OR recommendation OR guideline OR recommendations OR guidelines OR assessment OR assessments OR criteria OR criterias OR criterion OR "decision making" OR "decision-making" OR "decision point")) AND (ti(patient OR patients OR "patient advisory" OR "patient advocacy" OR "patient advocate" OR "patient association" OR "patient associations" OR "patient organization" OR "patient organisation" OR "patient organizations" OR "patient organisations" OR "health technology assessment body" OR "health technology assessment institution" OR "HTA body" OR "HTA institution" OR "reimbursement body" OR "national reimbursement body" OR "national reimbursement institution" OR "reimbursement institution" OR "reimbursement agency" OR regulatory OR "ministry of health" OR "regulatory body" OR "regulatory bodies" OR "regulator" OR EMA OR "European Medicines Agency" OR FDA OR "Food and Drug Administration" OR "policy maker" OR "Health Planning Organization" OR "Health Planning Organizations" OR "Health Planning Organisation" OR "Health Planning Organisations" OR "drug industry" OR "Medical device industry" OR "pharmaceutical industry" OR "pharmaceutical company" OR "pharmaceutical companies" OR "pharmaceutical sector" OR physician OR physicians OR clinician OR clinicians OR "health care professional" OR "health care professionals" OR "healthcare professional" OR "healthcare professionals" OR "health care provider" OR "health care providers" OR "healthcare provider" OR "healthcare providers" OR doctor OR doctors OR caregiver OR caregivers OR caretaker OR caretakers OR academic OR academics OR academician OR academicians OR researcher OR researchers) OR ab(patient OR patients OR "patient advisory" OR "patient advocacy" OR "patient advocate" OR "patient association" OR "patient associations" OR "patient organization" OR "patient organisation" OR "patient organizations" OR "patient organisations" OR "health technology assessment body" OR "health technology assessment institution" OR "HTA body" OR "HTA institution" OR "reimbursement body" OR "national reimbursement body" OR "national reimbursement institution" OR "reimbursement institution" OR "reimbursement agency" OR regulatory OR "ministry of health" OR "regulatory body" OR "regulatory bodies" OR "regulator" OR EMA OR "European Medicines Agency" OR FDA OR "Food and Drug Administration" OR "policy maker" OR "Health Planning Organization" OR "Health Planning Organizations" OR "Health Planning Organisation" OR "Health Planning Organisations" OR "drug industry" OR "Medical device industry" OR "pharmaceutical industry" OR "pharmaceutical company" OR "pharmaceutical companies" OR "pharmaceutical sector" OR physician OR physicians OR clinician OR clinicians OR "health care professional" OR "health care professionals" OR "healthcare professional" OR "healthcare professionals" OR "health care provider" OR "health care providers" OR "healthcare provider" OR "healthcare providers" OR doctor OR doctors OR caregiver OR caregivers OR caretaker OR caretakers OR academic OR academics OR academician OR academicians OR researcher OR researchers)) AND (ti("patients preference" OR "patients preferences" OR "preference of patients" OR "preferences of patients" OR "patient preference" OR "patient preferences" OR "preference of a patient" OR "preference of the patient" OR "preferences of a patient" OR "preferences of the patient") OR ab("patients preference" OR "patients preferences" OR "preference of patients" OR "preferences of patients" OR "patient preference" OR "patient preferences" OR "preference of a patient" OR "preference of the patient" OR "preferences of a patient" OR "preferences of the patient")) AND (ti("elicitation methods" OR method OR methodology OR empirical OR "qualitative method" OR qualitative OR "quantitative method" OR quantitative OR technique OR techniques OR Methodology OR Measuring OR measurement OR measurements OR assessment OR assessments OR inclusion OR including OR include OR incorporate OR incorporating OR incorporation OR involving OR involvement OR involve) OR ab("elicitation methods" OR method OR methodology OR empirical OR "qualitative method" OR qualitative OR "quantitative method" OR quantitative OR technique OR techniques OR Methodology OR Measuring OR measurement OR measurements OR assessment OR assessments OR inclusion OR including OR include OR incorporate OR incorporating OR incorporation OR involving OR involvement OR involve)) AND (ti("life cycle of a drug" OR "life cycle of a medical device" OR "medical device life cycle" OR "lifecycle of a drug" OR "lifecycle of a medical device" OR "medical device lifecycle" OR "drug life cycle" OR "drug lifecycle" OR "drug development" OR "medical device development" OR "development of drugs" OR "development of a drug" OR "development of medical devices" OR "development of a medical device" OR "benefit and risk" OR "risk and benefit" OR benefit-risk OR risk-benefit OR reimbursement OR "drug research" OR "medical device research" OR "clinical trials" OR "clinical trial" OR "health technology assessment" OR "health technology assessments") OR ab("life cycle of a drug" OR "life cycle of a medical device" OR "medical device life cycle" OR "lifecycle of a drug" OR "lifecycle of a medical device" OR "medical device lifecycle" OR "drug life cycle" OR "drug lifecycle" OR "drug development" OR "medical device development" OR "development of drugs" OR "development of a drug" OR "development of medical devices" OR "development of a medical device" OR "benefit and risk" OR "risk and benefit" OR benefit-risk OR risk-benefit OR reimbursement OR "drug research" OR "medical device research" OR "clinical trials" OR "clinical trial" OR "health technology assessment" OR "health technology assessments"))) NOT (ti("shared decision making" OR "shared decision-making" OR monitoring OR biomarker OR biomarkers) OR ab("shared decision making" OR "shared decision-making" OR monitoring OR biomarker OR biomarkers))) AND (la.exact("ENG") AND pd(20110101-20161231))

*Guidelines International Network*

“patient preferences”
