# Supplementary material for: Opportunities and challenges for the inclusion of patient preferences in the medical product life cycle: a systematic review
Source: BMC Med Inform Decis Mak. 2019 Oct 4;19:189. doi: 10.1186/s12911-019-0875-z (PMC6778383; doi:10.1186/s12911-019-0875-z)
Supplement: Supplementary file 2 — Included literature (DOCX 193 kb) [file 12911_2019_875_MOESM2_ESM.docx]

**Additional file 2: included literature**

|  | **Reference** | **Literature type** | **Stakeholder perspective^[[1]](#footnote-2)^** | **Decision-making context(s) described** |
| --- | --- | --- | --- | --- |
| 1 | FDA guidance [1] | Regulatory document | Regulatory authority | BRA |
| 2 | EMA report [2] | Regulatory document | Regulatory authority | BRA |
| 3 | KCE report [3] | HTA report | HTA body | HTA/reimbursement |
| 4 | IQWiG report [4] | HTA report | HTA body | HTA/reimbursement |
| 5 | Weernink MGM, 2014 [5] | Systematic review | Academic | BRA + HTA/reimbursement |
| 6 | Gutknecht M, 2016 [6] | Systematic review | Academic | HTA/reimbursement |
| 7 | Marsh K, 2017 [7] | Systematic review | Industry | BRA+ HTA/reimbursement |
| 8 | Brooker AS, 2013 [8] | Systematic review | HTA body representative | HTA/reimbursement |
| 9 | Irony T, 2016 [9] | Review | Regulator | BRA |
| 10 | Mühlbacher AC, 2013 [10] | Review | Academic | BRA + HTA/reimbursement + ITD + CPG |
| 11 | Johnson FR, 2016 [11] | Review | Academic | BRA |
| 12 | Martin-Fernandez J, 2014 [12] | Review | Academic | HTA/reimbursement |
| 13 | Mühlbacher AC, 2016 [13] | Review | Academic | BRA + HTA/reimbursement |
| 14 | Mühlbacher AC, 2015 [14] | Review | Academic | BRA + HTA/reimbursement |
| 15 | Puhan MA, 2012 [15] | Review | Academic | BRA |
| 16 | Stewart KD, 2016 [16] | Review | Academic | IPDM |
| 17 | Evers P, 2016 [17] | Review | Patient organization representative | IPDM + BRA + HTA/reimbursement |
| 28 | Ho MP, 2016 [18] | Review | Regulator | IPDM + BRA |
| 19 | Mott DJ, 2016 [19] | Review | Academic | BRA + HTA/reimbursement |
| 20 | Chaudhuri SE, 2018 [20] | Review | Academic | IPDM |
| 21 | Marsh K, 2018 [21] | Review | Industry | BRA+ HTA/reimbursement |
| 22 | Mühlbacher AC, 2016 [22] | Review | Academic | IPDM + BRA + HTA/reimbursement |
| 23 | Janssen EM, 2017 [23] | Review | Academic | BRA + HTA/reimbursement |
| 24 | Hauber B, 2013 [24] | Review | Academic | BRA |
| 25 | Tervonen T, 2017 [25] | Review | Industry | BRA |
| 26 | Utens C, 2015 [26] | Original research | Academic | HTA/reimbursement + CPG |
| 27 | Bridges JFP, 2014 [27] | Original research | Academic | BRA |
| 28 | Chow RD, 2014 [28] | Original research | Academic | IPDM |
| 29 | Danner M, 2011 [29] | Original research | Academic | HTA/reimbursement |
| 30 | Ho MP, 2015 [30] | Original research | Academic | BRA |
| 31 | Hollin IL,2016 [31] | Original research | Academic | BRA |
| 32 | Hummel MJM, 2012 [32] | Original research | Academic | HTA/reimbursement |
| 33 | Ijzerman MJ, 2012 [33] | Original research | Academic | HTA/reimbursement |
| 34 | Mol PG, 2015 [34] | Original research | Academic | BRA |
| 35 | Postmus D, 2016 [35] | Original research | Academic | BRA |
| 36 | Roy AN, 2015 [36] | Original research | Academic | IPDM + HTA/reimbursement |
| 37 | Morel T, 2016 [37] | Original research | Academic | BRA |
| 38 | Peay HL, 2014 [38] | Original research | Patient organization representative | BRA |
| 39 | Milovanovic S, 2017 [39] | Original research | Academic | HTA/reimbursement |
| 40 | Mühlbacher AC, 2016 [40] | Original research | Academic | ITD + BRA + HTA/reimbursement |
| 41 | Muhlbacher AC, 2017 [41] | Original research | Academic | BRA + HTA/reimbursement |
| 42 | Collison KA, 2018 [42] | Original research | Industry | IPDM |
| 43 | Janssen EM, 2017 [43] | Original research | Academic | ITD + BRA |
| 44 | Janssen IM, 2016 [44] | Original research | Academic | HTA/reimbursement |
| 45 | Kievit W, 2017 [45] | Original research | Academic | HTA/reimbursement |
| 46 | Stamuli E, 2017 [46] | Original research | Academic | IPDM |
| 47 | von Arx LB, 2017 [47] | Original research | Academic | BRA |
| 48 | Avila M, 2015 [48] | Original research | Academic | HTA/reimbursement |
| 49 | Medical Device Innovation Consortium (MDIC) report, 2015 [49] | Project report | / | IPDM + BRA |
| 50 | PROTECT report 'Recommendations for Patient and Public Involvement in the assessment of benefit and risk of medicines', 2013 [50] | Project report | / | BRA |
| 51 | Biotechnology Innovation Organization and Parent Project Muscular Dystrophy report, 2016 [51] | Project report | / | BRA |
| 52 | EUPATI report, 2016 [52] | Project report | / | HTA/reimbursement |
| 53 | Avalere and Milken Institute report [53] | Project report | / | ITD + BRA |
| 54 | PROTECT report 'Recommendations for the methodology and visualisation techniques to be used in the assessment of benefit and risk of medicines.' [54] | Project report | / | BRA |
| 55 | PROTECT report 'Review of methodologies for benefit and risk assessment of medication.' [55] | Project report | / | BRA |
| 56 | EMA workshop report [56] | Workshop report | Regulatory authority | BRA + HTA/reimbursement |
| 57 | FasterCures workshop report [57] | Workshop report | Patient organization | ITD + BRA |
| 58 | DIA workshop report [58] | Workshop report | Industry | IPDM + BRA |
| 59 | Smith MY, 2016 [59] | Perspective article | Industry | IPDM + BRA |
| 60 | Dirksen CD, 2014 [60] | Perspective article | Academic | HTA/reimbursement |
| 61 | Eichler HG, 2013 [61] | Perspective article | Regulator | BRA |
| 62 | Egbrink MO, 2014 [62] | Perspective article | Academic | BRA + HTA/reimbursement |
| 63 | Eichler HG, 2012 [63] | Perspective article | Regulator | BRA |
| 64 | van Til JA, 2014 [64] | Perspective article | Academic | BRA |
| 65 | Hunter NL, 2016 [65] | Perspective article | Regulator | BRA |
| 66 | Mühlbacher AC, 2017 [66] | Perspective article | Academic | BRA + HTA/reimbursement |
| 67 | Craig BM, 2017 [67] | Perspective article | Academic | ITD + BRA + HTA/reimbursement |
| 68 | Johnson FR, 2017 [68] | Perspective article | Academic | BRA |
| 69 | Mott DJ, 2018 [69] | Perspective article | Academic | HTA/reimbursement |
| 70 | Wolka AM, 2017 [70] | Other | Industry | BRA |
| 71 | Marsh K, 2016 [71] | Other | Industry | IPDM + BRA + HTA/reimbursement |
| 72 | Pisa G, 2015 [72] | Other | Academic | BRA + HTA/reimbursement |

**Legend**

BRA= benefit-risk assessment

HTA= health technology assessment

IPDM= industry processes and decision-making

CPG= clinical practice guideline development

ITD= individual treatment decision-making

**Reference list**

1. Patient Preference Information – Voluntary Submission, Review in Premarket Approval Applications, Humanitarian Device Exemption Applications, and De Novo Requests, and Inclusion in Decision Summaries and Device Labeling: Guidance for Industry, Food and Drug Administration Staff, and Other Stakeholders. U.S. Department of Health and Human Services, Food and Drug Administration, Center for Devices and Radiological Health and Center for Biologics Evaluation and Research; 2016.

2. The patient's voice in the evaluation of medicines. European Medicines Agency, Stakeholders and Communication Division; 2013. Report No.: EMA/607864/2013.

3. Christiaens W, Kohn L, Léonard C, Denis A, Daue F, Cleemput I. Models for citizen and patient involvement in health care policy - Part I: exploration of their feasibility and acceptability. Health Services Research (HSR). Brussels: Belgian Health Care Knowledge Centre (KCE); 2012. Report No.: KCE Reports 195C. D/2012/10.273/105.

4. Choice-based Conjoint Analysis – pilot project to identify, weight, and prioritize multiple attributes in the indication “hepatitis C”. Institute for Quality and Efficiency in Health Care (IQWiG); 2014. Report No.: GA10-03.

5. Weernink MGM, Janus SIM, van Til JA, Raisch DW, van Manen JG, Ijzerman MJ. A Systematic Review to Identify the Use of Preference Elicitation Methods in Healthcare Decision Making. Pharmaceutical Medicine. 2014;28(4):175-85.

6. Gutknecht M, Schaarschmidt ML, Herrlein O, Augustin M. A systematic review on methods used to evaluate patient preferences in psoriasis treatments. Journal of the European Academy of Dermatology and Venereology. 2016.

7. Marsh K, Caro JJ, Hamed A, Zaiser E. Amplifying Each Patient’s Voice: A Systematic Review of Multi-criteria Decision Analyses Involving Patients. Applied Health Economics and Health Policy. 2017;15(2):155-62.

8. Brooker AS, Carcone S, Witteman W, Krahn M. Quantitative patient preference evidence for health technology assessment: A case study. International journal of technology assessment in health care. 2013;29(3):290-300.

9. Irony T, Ho M, Christopher S, Levitan B. Incorporating Patient Preferences into Medical Device Benefit-Risk Assessments. Statistics in Biopharmaceutical Research. 2016;8(3):230-6.

10. Muhlbacher AC, Juhnke C. Patient preferences versus physicians' judgement: does it make a difference in healthcare decision making? Appl Health Econ Health Policy. 2013;11(3):163-80.

11. Johnson FR, Zhou M. Patient Preferences in Regulatory Benefit-Risk Assessments: A US Perspective. Value in Health. 2016;19(6):741-5.

12. Martin-Fernandez J, Polentinos-Castro E, del Cura-Gonzalez MI, Ariza-Cardiel G, Abraira V, Gil-LaCruz AI, et al. Willingness to pay for a quality-adjusted life year: an evaluation of attitudes towards risk and preferences. BMC Health Serv Res. 2014;14:287.

13. Mühlbacher AC, Juhnke C, Beyer AR, Garner S. Patient-Focused Benefit-Risk Analysis to Inform Regulatory Decisions: The European Union Perspective. Value in Health. 2016;19(6):734-40.

14. Mühlbacher AC. Patient-centric HTA: Different strokes for different folks. Expert Review of Pharmacoeconomics and Outcomes Research. 2015;15(4):591-7.

15. Puhan MA, Singh S, Weiss CO, Varadhan R, Boyd CM. A framework for organizing and selecting quantitative approaches for benefit-harm assessment. BMC medical research methodology. 2012;12:173.

16. Stewart KD, Johnston JA, Matza LS, Curtis SE, Havel HA, Sweetana SA, et al. Preference for pharmaceutical formulation and treatment process attributes. Patient preference and adherence. 2016;10:1385-99.

17. Evers P, Greene L, Ricciardi M. The importance of early access to medicines for patients suffering from rare diseases. Regulatory Rapporteur. 2016;13:5-8.

18. Ho M, Saha A, McCleary KK, Levitan B, Christopher S, Zandlo K, et al. A Framework for Incorporating Patient Preferences Regarding Benefits and Risks into Regulatory Assessment of Medical Technologies. Value in Health. 2016;19(6):746-50.

19. Mott DJ, Najafzadeh M. Whose preferences should be elicited for use in health-care decision-making? A case study using anticoagulant therapy. Expert Review of Pharmacoeconomics and Outcomes Research. 2016;16(1):33-9.

20. Chaudhuri SE, Ho MP, Irony T, Sheldon M, Lo AW. Patient-centered clinical trials. Drug discovery today. 2018;23(2):395-401.

21. Marsh K, Caro JJ, Zaiser E, Heywood J, Hamed A. PATIENT-CENTERED DECISION MAKING: LESSONS from MULTI-CRITERIA DECISION ANALYSIS for QUANTIFYING PATIENT PREFERENCES. International journal of technology assessment in health care. 2018;34(1):105-10.

22. Mühlbacher AC, Kaczynski A. Making Good Decisions in Healthcare with Multi-Criteria Decision Analysis: The Use, Current Research and Future Development of MCDA. Applied Health Economics and Health Policy. 2016;14(1):29-40.

23. Janssen EM, Marshall DA, Hauber AB, Bridges JFP. Improving the quality of discrete-choice experiments in health: how can we assess validity and reliability? Expert Review of Pharmacoeconomics and Outcomes Research. 2017;17(6):531-42.

24. Brett Hauber A, Fairchild AO, Reed Johnson F. Quantifying benefit-risk preferences for medical interventions: an overview of a growing empirical literature. Appl Health Econ Health Policy. 2013;11(4):319-29.

25. Tervonen T, Gelhorn H, Sri Bhashyam S, Poon JL, Gries KS, Rentz A, et al. MCDA swing weighting and discrete choice experiments for elicitation of patient benefit-risk preferences: a critical assessment. Pharmacoepidemiology and drug safety. 2017;26(12):1483-91.

26. Utens C., Dirksen C., van der Weijden T., M. AJ. How to integrate research evidence on patient preferences in pharmaceutical coverage decisions and clinical practice guidelines: A qualitative study among Dutch stakeholders. Health Policy. 2015;120(1):120-8.

27. Bridges JFP, Paly VF, Barker E, Kervitsky D. Identifying the Benefits and Risks of Emerging Treatments for Idiopathic Pulmonary Fibrosis: A Qualitative Study. The patient. 2014;8(1):85-92.

28. Chow RD, Wankhedkar KP, Mete M. Patients' preferences for selection of endpoints in cardiovascular clinical trials. Journal of community hospital internal medicine perspectives. 2014;4.

29. Danner M, Hummel JM, Volz F, Van Manen JG, Wiegard B, Dintsios CM, et al. Integrating patients' views into health technology assessment: Analytic hierarchy process (AHP) as a method to elicit patient preferences. International journal of technology assessment in health care. 2011;27(4):369-75.

30. Ho MP, Gonzalez JM, Lerner HP, Neuland CY, Whang JM, McMurry-Heath M, et al. Incorporating patient-preference evidence into regulatory decision making. Surgical endoscopy. 2015;29(10):2984-93.

31. Hollin IL, Peay H, Apkon SD, Bridges J. Patient-centered benefit-risk assessment in Duchenne Muscular Dystrophy. Muscle & nerve. 2016.

32. Hummel MJM, Volz F, Van Manen JG, Danner M, Dintsios CM, Ijzerman MJ, et al. Using the analytic hierarchy process to elicit patient preferences: Prioritizing multiple outcome measures of antidepressant drug treatment. The patient. 2012;5(4):225-37.

33. Ijzerman MJ, Van Til JA, Bridges JFP. A comparison of analytic hierarchy process and conjoint analysis methods in assessing treatment alternatives for stroke rehabilitation. The patient. 2012;5(1):45-56.

34. Mol PG, Arnardottir AH, Straus SM, de Graeff PA, Haaijer-Ruskamp FM, Quik EH, et al. Understanding drug preferences, different perspectives. Br J Clin Pharmacol. 2015;79(6):978-87.

35. Postmus D, Mavris M, Hillege HL, Salmonson T, Ryll B, Plate A, et al. Incorporating patient preferences into drug development and regulatory decision making: Results from a quantitative pilot study with cancer patients, carers, and regulators. Clinical Pharmacology and Therapeutics. 2016;99(5):548-54.

36. Roy AN, Madhavan SS, Lloyd A. A Discrete Choice Experiment to Elicit Patient Willingness to Pay for Attributes of Treatment-Induced Symptom Relief in Comorbid. Insomnia. Manag Care. 2015;24(4):42-8.

37. Morel T, Ayme S, Cassiman D, Simoens S, Morgan M, Vandebroek M. Quantifying benefit-risk preferences for new medicines in rare disease patients and caregivers. Orphanet J Rare Dis. 2016;11(1):70.

38. Peay HL, Hollin I, Fischer R, Bridges JF. A community-engaged approach to quantifying caregiver preferences for the benefits and risks of emerging therapies for Duchenne muscular dystrophy. Clinical therapeutics. 2014;36(5):624-37.

39. Milovanovic S, Scaldaferri F, Canarecci S, Kheiraoui F, Ciancarella G, de Waure C, et al. Therapy experiences and preferences among patients with anemia: Results of a cross-sectional survey among Italian patients with inflammatory bowel disease. Digestive and Liver Disease. 2017;49(10):1098-103.

40. Mühlbacher A, Bethge S. What matters in type 2 diabetes mellitus oral treatment? A discrete choice experiment to evaluate patient preferences. European Journal of Health Economics. 2016;17(9):1125-40.

41. Mühlbacher AC, Bridges JF, Bethge S, Dintsios CM, Schwalm A, Gerber-Grote A, et al. Preferences for antiviral therapy of chronic hepatitis C: a discrete choice experiment. The European journal of health economics : HEPAC : health economics in prevention and care. 2017;18(2):155-65.

42. Collison KA, Patel P, Preece AF, Stanford RH, Sharma RK, Feldman G. A Randomized Clinical Trial Comparing the ELLIPTA and HandiHaler Dry Powder Inhalers in Patients With COPD: Inhaler-Specific Attributes and Overall Patient Preference. COPD: Journal of Chronic Obstructive Pulmonary Disease. 2018;15(1):46-50.

43. Janssen EM, Longo DR, Bardsley JK, Bridges JF. Education and patient preferences for treating type 2 diabetes: a stratified discrete-choice experiment. Patient preference and adherence. 2017;11:1729-36.

44. Janssen IM, Scheibler F, Gerhardus A. Importance of hemodialysis-related outcomes: Comparison of ratings by a self-help group, clinicians, and health technology assessment authors with those by a large reference group of patients. Patient preference and adherence. 2016;10:2491-500.

45. Kievit W, Tummers M, Van Hoorn R, Booth A, Mozygemba K, Refolo P, et al. Taking patient heterogeneity and preferences into account in health technology assessments. International journal of technology assessment in health care. 2017;33(5):562-9.

46. Stamuli E, Torgerson D, Northgraves M, Ronaldson S, Cherry L. Identifying the primary outcome for a randomised controlled trial in rheumatoid arthritis: the role of a discrete choice experiment. Journal of foot and ankle research. 2017;10:57.

47. von Arx LB, Johnson FR, Morkbak MR, Kjaer T. Be Careful What You Ask For: Effects of Benefit Descriptions on Diabetes Patients' Benefit-Risk Tradeoff Preferences. Value in health : the journal of the International Society for Pharmacoeconomics and Outcomes Research. 2017;20(4):670-8.

48. Avila M, Becerra V, Guedea F, Suarez JF, Fernandez P, Macias V, et al. Estimating preferences for treatments in patients with localized prostate cancer. International journal of radiation oncology, biology, physics. 2015;91(2):277-87.

49. Medical Device Innovation Consortium (MDIC) Patient Centered Benefit-Risk Project Report: A Framework for Incorporating Information on Patient Preferences regarding Benefit and Risk into Regulatory Assessments of New Medical Technology. Medical Device Innovation Consortium; 2015.

50. Hockley K, Ashby D, Das S, Hallgreen C, Mt-Isa S, Waddingham E, et al. Patient and Public Involvement Report: Recommendations for Patient and Public Involvement in the assessment of benefit and risk of medicines. Innovative Medicines Initiative, Pharmacoepidemiological Research on Outcomes of Therapeutics by a European ConsorTium (PROTECT), Benefit-Risk Group; 2013.

51. Selig WKD. Key considerations for developing & integrating patient perspectives in drug development: Examination of the duchenne case study. Biotechnology Innovation Organization and Parent Project Muscular Dystrophy; 2016.

52. Patient involvement in the HTA decision-making process. Innovative Medicines Initiative, European Patients' Academy on Therapeutic Innovation (EUPATI); 2016.

53. Patient-Perspective Value Framework (PPVF): Draft Methodology. Avalere and Milken Institute, FasterCures; 2016.

54. Hughes D, Waddingham EAJ, Mt-Isa S, Goginsky A, Chan E, Downey G, et al. Recommendations for the methodology and visualisation techniques to be used in the assessment of benefit and risk of medicines. Innovative Medicines Initiative, Pharmacoepidemiological Research on Outcomes of Therapeutics by a European ConsorTium (PROTECT), Benefit-Risk Group; 2013.

55. Mt-Isa S, Wang N, Hallgreen CE, Callréus T, Genov G, Hirsch I, et al. Review of methodologies for benefit and risk assessment of medication. Innovative Medicines Initiative, Pharmacoepidemiological Research on Outcomes of Therapeutics by a European ConsorTium (PROTECT); 2013.

56. European Medicines Agency. Regulatory and methodological standards to improve benefit-risk evaluation of medicines 2014 [6]. Available from: <http://www.ema.europa.eu/docs/en_GB/document_library/Report/2014/04/WC500165803.pdf>.

57. Partnering with patients on value, coverage, and reimbursement: A summary from the June 11, 2015 FasterCures workshop. Milken Institute, FasterCures; 2015.

58. Smith MY, Hammad TA, Metcalf M, Levitan B, Noel R, Wolka AM, et al. Patient Engagement at a Tipping Point—The Need for Cultural Change Across Patient, Sponsor, and Regulator Stakeholders: Insights From the DIA Conference, “Patient Engagement in Benefit Risk Assessment Throughout the Life Cycle of Medical Products”. Therapeutic Innovation and Regulatory Science. 2016;50(5):546-53.

59. Smith MY, Benattia I. The Patient’s Voice in Pharmacovigilance: Pragmatic Approaches to Building a Patient-Centric Drug Safety Organization. Drug Safety. 2016;39(9):779-85.

60. Dirksen CD. The use of research evidence on patient preferences in health care decision-making: issues, controversies and moving forward. Expert review of pharmacoeconomics & outcomes research. 2014;14(6):785-94.

61. Eichler HG, Bloechl-Daum B, Brasseur D, Breckenridge A, Leufkens H, Raine J, et al. The risks of risk aversion in drug regulation. Nature Reviews Drug Discovery. 2013;12(12):907-16.

62. Egbrink MO, M IJ. The value of quantitative patient preferences in regulatory benefit-risk assessment. Journal of market access & health policy. 2014;2.

63. Eichler HG, Abadie E, Baker M, Rasi G. Fifty years after thalidomide; what role for drug regulators? Br J Clin Pharmacol. 2012;74(5):731-3.

64. van Til JA, Ijzerman MJ. Why should regulators consider using patient preferences in benefit-risk assessment? PharmacoEconomics. 2014;32(1):1-4.

65. Nina LH, Center for D, Radiological Health USF, Drug Administration SSM, Kathryn MOC, Center for D, et al. Engaging Patients Across the Spectrum of Medical Product Development: View From the US Food and Drug Administration. JAMA. 2017;314(23):2499-500.

66. Mühlbacher AC, Johnson FR. Giving Patients a Meaningful Voice in European Health Technology Assessments: The Role of Health Preference Research. The patient. 2017;10(4):527.

67. Craig BM, Lancsar E, Muhlbacher AC, Brown DS, Ostermann J. Health Preference Research: An Overview. The patient. 2017;10(4):507-10.

68. Johnson FR, Beusterien K, Ozdemir S, Wilson L. Giving Patients a Meaningful Voice in United States Regulatory Decision Making: The Role for Health Preference Research. The patient. 2017;10(4):523-6.

69. Mott DJ. Incorporating Quantitative Patient Preference Data into Healthcare Decision Making Processes: Is HTA Falling Behind? Patient. 112018. p. 249-52.

70. Wolka AM, Fairchild AO, Reed SD, Anglin G, Johnson FR, Siegel M, et al. Effective Partnering in Conducting Benefit-Risk Patient Preference Studies: Perspectives From a Patient Advocacy Organization, a Pharmaceutical Company, and Academic Stated-Preference Researchers. Therapeutic Innovation and Regulatory Science. 2017.

71. Marsh K. Incorporating Patient Preferences into Product Development and Value Communication: Why, When and How? The Evidence Forum: A Discourse on Value. 2016;May:38-41.

72. Pisa G. A Step Towards Patient-Centricity: Analysis of HTA Requirements for Patient Preference Data Collection in Germany. Kantar Health. 2015;September:1-6.

1. *To define the type of stakeholder upon which the opinion in a particular document is based, each document was assigned to a certain stakeholder group: for primary research articles, (systematic) reviews and perspective articles, the affiliation cited of the first author was used to assign a stakeholder perspective. For regulatory documents, the regulatory agency perspective was assigned. HTA reports were assigned to the HTA body perspective. For project reports, since those are written from a multitude of stakeholder perspectives, they could not be assigned to a specific stakeholder perspective.* [↑](#footnote-ref-2)
