# Supplementary material for: Opportunities and challenges for the inclusion of patient preferences in the medical product life cycle: a systematic review
Source: BMC Med Inform Decis Mak. 2019 Oct 4;19:189. doi: 10.1186/s12911-019-0875-z (PMC6778383; doi:10.1186/s12911-019-0875-z)
Supplement: Supplementary file 3 — Coding tree (DOCX 96 kb) [file 12911_2019_875_MOESM3_ESM.docx]

**Additional file 3: coding tree**

1. General considerations
   - Desires
     - Value of PP in all stages of the medical product life cycle
     - One PP method to inform both Benefit-Risk Assessment and Health Technology Assessment
   - Expectations
   - Concerns
   - Requirements
     - Interaction between stakeholders
2. Industry processes and decision-making
   - Desires
     - Value of PP in industry processes and decision-making
   - Expectations
   - Concerns
     - Methodological and operational concerns
       - Sample characteristics
       - Recruitment
       - Feasibility
   - Requirements
     - Methodological and operational requirements
       - Type of PP method
         - Feasibility
       - Type of patient preferences
         - Reliability, validity and generalizability
       - Sample characteristics
3. Benefit-risk assessment (BRA)
   - Desires
     - Value of PP in BRA
   - Expectations
   - Concerns
     - Methodological and operational concerns
       - Sample characteristics
       - Recruitment
       - Feasibility
     - Interpretation and uptake of PP
       - Aligning PP with clinical trial results
   - Requirements
     - Methodological and operational requirements
       - Type of PP method
         - Feasibility
       - Type of patient preferences
         - Reliability, validity and generalizability

- Sample characteristics

1. Health technology assessment (HTA)
   - Desires
     - Value of PP in HTA
   - Expectations
   - Concerns
     - Methodological and operational concerns
       - Sample characteristics
       - Recruitment
       - Feasibility
     - Interpretation and uptake of PP
       - Aligning PP with other data
   - Requirements
     - Methodological and operational requirements
       - Type of PP method
         - Feasibility
       - Type of patient preferences
         - Reliability, validity and generalizability
         - Generic versus disease-specific information
       - Sample characteristics
